# Excessive scleral shrinkage, rather than choroidal thickening, is a major contributor to the development of hypotony maculopathy after trabeculectomy

**DOI:** 10.1371/journal.pone.0191862

**Published:** 2018-01-26

**Authors:** Mari Sakamoto, Yoshiko Matsumoto, Sotaro Mori, Kaori Ueda, Yukako Inoue, Takuji Kurimoto, Akiyasu Kanamori, Yuko Yamada, Makoto Nakamura

**Affiliations:** Department of Surgery, Division of Ophthalmology, Kobe University Graduate School of Medicine, Kobe, Japan; Boston University School of Medicine, UNITED STATES

## Abstract

**Purpose:**

We previously reported that eyes with hypotony maculopathy (HM) after trabeculectomy (TLE) exhibited more reduction of axial length (AL) than those without HM, suggesting that inward collapse of the scleral wall may contribute to the development of HM after TLE. However, we did not evaluate change in choroidal thickness (CT), which could influence AL measures. We compared the magnitude and rate of AL and CT changes in eyes with and without HM by simultaneously measuring these parameters before and after TLE.

**Methods:**

We enrolled 77 eyes of 77consecutive patients with glaucoma, who underwent TLE between March 2014 and March 2016. Intraocular pressure (IOP), central corneal thickness, keratometry, AL, and CT were measured pre- and postoperatively, up to 6 months. These biometrics were compared in eyes with and without HM.

**Results:**

The 14 patients who developed HM were significantly younger than those who did not. The eyes with HM exhibited significantly reduced AL (2.8%) compared to those without HM (0.7%). There was no significant difference in CT change between the two groups. The rate of AL reduction was significantly correlated with age, postoperative IOP, and preoperative AL. Post-adjustment logistic regression analysis revealed that eyes with AL reduction rate ≥ 2% had 11.67 higher risk for developing HM (95% confidence interval, 1.28–106.6; *P* = 0.03).

**Conclusions:**

AL reduction rates ≥ 2% were significantly associated with HM. Excessive reduction in AL, which was seen in eyes with HM, was not an artificial measure resulting from choroidal thickening but rather reflected reductions in the anterior-posterior diameter of the eyeball. Inward collapse of the scleral wall leads to redundancy of the chorioretinal tissue, contributing to the development of HM after TLE.

## Introduction

Hypotony maculopathy (HM) is a sight-threatening complication after trabeculectomy (TLE). It is characterized by chorioretinal folds, vascular tortuosity, and/or optic disc swelling. Delayed treatment of HM can result in vision loss because of permanent structural changes to the retina and choroid. The incidence of HM after TLE has been reported to be up to 20% [1, 2]. Male gender, myopia, young age, primary filtering surgery, and adjunctive use of antifibrotic agents, such as 5-fluorouracil and mitomycin C (MMC), are the reported risk factors for developing HM [1, 2].

Regarding the mechanisms of development of HM after TLE, Gass associated scleral biomechanical properties with the development of chorioretinal folds induced by hypotony[3]. In other words, he speculated that hypotony-induced scleral wall collapse causes redundancy of the retina and choroid tissue. However, there is no confirmative evidence that supports Gass’ theory. A recent study using swept source optical coherence tomography (OCT) demonstrated that IOP lowering after trabeculectomy induced scleral shrinkage in eyes with pathologic myopia. However, that study did not evaluate whether or not excessive scleral shrinkage was associated with the development of hypotony maculopathy[4].

Accumulating evidence showed that axial length (AL) is significantly reduced after TLE; however, the magnitude was reportedly low [5–10]. In contrast, we previously reported that at 4 weeks after MMC-augmented TLE, eyes with HM showed approximately 4-fold greater reduction of AL than those without HM [11], suggesting that excessive scleral wall shrinkage contributes to the development of HM after TLE. However, that study used partial coherence laser interferometry to measure AL. This method defines AL as the distance from the corneal surface to the retinal pigment epithelium and does not measure choroidal thickness (CT). Given that the CT thickens after TLE [5–7, 10], greater AL reduction in eyes with HM may result from excessive choroidal thickening following TLE. Moreover, our previous retrospective study only included eyes with hypotony, arbitrarily defined as an intraocular pressure (IOP) below 6.5 mmHg (less than 3 × standard deviation of normal IOP distribution). This may have produced an inclusion bias. Because of these methodological limitations, our previous study could not conclude whether or not scleral wall shrinkage was responsible for HM development after TLE.

The aim of the current study was to test if excessive reductions in AL, in eyes with HM after TLE, were due to scleral wall collapse, rather than choroidal thickening. To elucidate this issue, we consecutively enrolled patients undergoing initial TLE, and simultaneously measured AL and CT, before and after surgery. We then estimated the relative risk of excessive AL reduction for developing HM.

## Materials and methods

### Study design and ethics statement

This was a cross-sectional, observational cohort study of consecutive patients undergoing glaucoma surgery at Kobe University Hospital. The study protocol was approved by the Institutional Review Board of Kobe University (UMIN000006900 and UMIN000011069) and adhered to the tenets of the Declaration of Helsinki. We obtained written informed consent from each patient after explaining the nature of the study.

### Subject selection and enrollment criteria

Subjects with open angle glaucoma were consecutively enrolled when they underwent initial TLE, with an intraoperative 3-minute application of 0.04% MMC, between March 2014 and March 2016. Subjects who underwent TLE combined with cataract surgery were also included. Only one eye per subject was examined for the purposes of this study. When both eyes underwent TLE, the first eye was examined.

Exclusion criteria were angle closure glaucoma, congenital glaucoma, secondary glaucoma (except for exfoliation glaucoma), a history of previous intraocular surgeries (except for uncomplicated cataract surgery), uveitis, choroidal and retinal diseases, and poor quality of OCT images for correct measurement of CT.

### Tested parameters and optical coherence tomography measurement

All subjects underwent ophthalmologic examinations preoperatively, and at 2 weeks, one month, 3 months, and 6 months after TLE. Examinations included slit-lamp biomicroscopy, visual acuity, refraction, keratometry (Auto ref-keratometer RC-5000; TOMEY, Nagoya, Japan), IOP (Goldmann applanation tonometry), central corneal thickness (Noncon Robo®; Konan Medial, Nishinomiya, Japan), AL (IOLMaster; Carl Zeiss Meditec; Dublin, CA, USA), fundus photography, and fundus imaging using Cirrus spectral-domain OCT (Carl Zeiss Meditec) and Spectralis (Heidelberg Instruments, Heidelberg, Germany).

Cirrus OCT imaging was performed using the Macular Cube protocol for determining HM, as described previously[11]. We defined HM as the presence of vascular tortuosity and chorioretinal folds on ophthalmoscopy and/or en face OCT images. The presence of optic disc swelling was not necessary for study inclusion. Two examiners (YY and AK), masked to patients’ clinical findings, analyzed the images to determine the presence of macular abnormalities. Subjects were divided into two groups based on the presence, or absence, of HM.

Spectralis OCT images, centered on the fovea, were obtained using an enhanced depth imaging mode for measuring CT. The macular region was scanned using a single 30° linear B-scan, made up of 1536 A-scans, centered on the fovea averaged 100 times.

Keratometry readings and refraction were entered to adjust optical magnification. After adjusting the B-scan scale to 1:1, and doubling the imaging size, a single observer (MS) manually measured the distance from the outer margin of the retinal pigment epithelium to the choroidal-scleral interface at four points (750μm superior, inferior, nasal, and temporal to the fovea), using a built-in linear measuring tool. We defined the average thickness of the four measurements as the patient’s CT. Only images with sufficient quality for identification of the outer margin of the retinal pigment epithelium and the choroidal-scleral interface were included.

### Statistical analysis

Study sample size was predetermined. Based on our previous study [11], the HM occurs in approximately one out of six TLE surgeries, correspondingly, we set the allocation ratio, for the presence and absence of HM, at 1:5. We set standard deviation for percent AL change 1.0% (in eyes without HM), and 2.5% (in eyes with HM), and the true difference in the mean of percent AL changes, between eyes with and without HM, as 2.5%. We required 12 patients with HM, and 59 patients without HM, in order to reject the null hypothesis that the population means of the percent AL change, between the eyes with and without HM, were equal to the Type II error (0.1; statistical power being 90%). The probability of Type I error associated with the test of this null hypothesis was 0.05.

Statistical analyses were performed using MedCalc (version 16.8.4, MedCalc Software, Mariakerte, Belgium) and SPSS Statistics (version 24, IBM Japan, Tokyo, Japan) with Type I error for significance set at *P* < 0.05.

To compare patient demographics between the two groups, we used unpaired t-tests or the Mann-Whitney U-test for continuous variables, depending on data distribution. For within group comparisons, we used paired t-tests or Wilcoxon tests. Comparisons of categorical variables were performed using Fisher’s exact probability test. Bivariate relationships were analyzed using Spearman’s rank correlation test and linear regression analysis. Propensity scores were calculated using a bivariate logistic regression model. A logistic regression model, with inverse probability weighting, was conducted using robust variance, as included in the generalized estimating equation model package of SPSS.

## Results

A total of 77 eyes, from 77 open angle patients with glaucoma who met all inclusion and exclusion criteria were enrolled in this study. This included 70 eyes with POAG, and seven eyes with exfoliation glaucoma. Sixty-nine eyes underwent TLE alone, while eight underwent TLE combined with cataract surgery. Fourteen out of 77 subjects developed HM, at a minimum of one time point, up to 6 months after TLE.

Table 1 summarizes demographic data and postoperative complications, in eyes with and without HM. Subjects with HM were significantly younger than those without HM. There were no differences in gender distribution, postoperative complications, or the number of times laser suture lysis was performed, between those with and without HM. Bleb leaks were halted within a day or two, either spontaneously or by suture-repair. Overfiltraion was defined by the slit-lamp biomicroscopic appearance of filtering bleb according to the Indiana bleb appearance grading scale [12] and an IOP level. In other words, an eye that exhibited the bleb appearance with moderate or high bleb height, a horizontal extent over 2 clock hours, and the IOP less than 6 mmHg were defined as having overfiltration.

**Table 1:** Demographic data and postoperative course of the two groups.

		HM(-) (n = 63)	HM(+) (N = 14)	*P*-value
Age(years)		68.9 ± 11 (40–88)	61.5 ± 11 (40–77)	0.02*
Male gender		34 (54.0%)	8 (57.1%)	1.00^†^
Number of laser suture lysis		0 (0 to 1)	0 (0 to 2)	0.79^‡^
Other complications	Choroidal detachment	16 (25.4%)	6 (42.9%)	0.21^†^
	Bleb leak	13 (20.6%)	2 (14.3%)	0.72^†^
	Over filtration	10 (15.9%)	2 (14.3%)	1.00^†^

HM, hypotony maculopathy

Data are expressed as mean ± standard deviation (range) for age and median (95% confidence interval) for the number for suture lysis.

*Independent sample t-test

^†^Fisher’s exact test

^‡^Mann-Whitney test

The time point of postoperative IOP troughs varied among individuals. We recorded the number of subjects who showed the trough IOP at 2 weeks, one month, and 3 months after the surgery was 43, 15, and 5 in the eyes without HM and 7, 6, and 1 in the eyes with HM. No one exhibited the lowest IOP at 6 months after surgery. Measurements obtained at the visit showing the trough IOP were selected for further analyses. If the IOP measurements were the same during multiple visits, the data at the earliest postoperative visit were chosen. HM, if detected at all, was always detected at the visit showing the trough IOP.

Table 2 shows preoperative and postoperative data at the visits with trough IOP, in both groups. In this study, the identification of the choroidal–scleral interface using Spectralis OCT at the postoperative visits was challenging in only 1 of the 77 eyes enrolled, which was excluded from the statistical analyses for choroidal thickness. Although the preoperative AL tended to be longer in eyes with HM, compared with those without HM, the difference was not statistically significant. Preoperative IOP, CT, central corneal thickness, and corneal curvature were not different between eyes with and without HM. There were significant changes in all variables, except central corneal thickness, pre- to postoperatively, in eyes with and without HM. IOP and AL were significantly reduced, the choroid was significantly thickened, and the cornea was significantly steepened, postoperatively, irrespective of the existence of HM. Postoperative IOP, in eyes with HM, was significantly lower than in those without HM; median trough IOPs were 5 mmHg (with HM) and 8 mmHg (without HM). There were no significant differences in other postoperative variables between the two groups. However, when the pre- and postoperative variables were compared, IOP and AL had significantly greater magnitude, and rate of reduction, in eyes with HM (16 mmHg IOP reduction and 2.8% AL reduction) compared to those without HM (10 mmHg IOP reduction and 0.7% AL reduction). There were no significant differences in postoperative changes in CT, central corneal thickness, and corneal radius after TLE, between eyes with and without HM.

**Table 2:** Pre-and postoperative data.

		Preoperative data	Postoperative data	*P*-value*	Magnitude of difference	Percent difference
**IOP (mmHg)**	HM(-)	18.0 (17.0 to 19.0)	8.0 (7.0 to 8.7)	<0.0001	−10.0 (−11.0 to −9.0)	−56.3 (−61.7 to 53.2)
	HM(+)	20.0 (16.9 to 25.3)	5.0 (3.5 to 6.9)	0.0002	−16.0 (−20.0 to −11.1)	−79.0 (−85.3 to 63.5)
	*P*-value^†^	0.21	0.006		0.0017	0.0001
**AL (mm)**	HM(-)	24.9 (24.5 to 25.4)	24.7 (24.3 to 25.2)	<0.0001	−0.18 (−0.22 to −0.14)	−0.7 (−0.9 to −0.6)
	HM(+)	26.4 (24.0 to 27.1)	25.8 (23.3 to 26.4)	0.0001	−0.70 (−1.18 to −0.52)	−2.8 (−4.7 to −1.9)
	*P*-value^†^	0.056	0.34		<0.0001	<0.0001
**CT (μm)**	HM(-)	154.5 (138.5 to 189.2)	184.3 (160.2 to 227.3)	<0.0001	31.4 (22.0 to 38.8)	22.2 (16.2 to 25.7)
	HM(+)	165 (95.1 to 253.8)	201.4 (132.3 to 290)	0.025	27.0 (9.87 to 40.94)	16.3 (6.5 to 30.1)
	*P*-value^†^	0.74	0.98		0.34	0.38
**CCT (μm)**	HM(-)	510.5 (499.0 to 526.2)	509.0 (491.4 to 525.0)	0.40	2.0 (−9.0 to 8.0)	0.4 (−1.9 to 1.5)
	HM(+)	516.5 (467.8 to 536.5)	523.0 (500.7 to 567.7)	0.33	13.5 (−15.1 to 23.3)	2.5 (−2.9 to 4.2)
	*P*-value^†^	0.94	0.23		0.29	0.29
**K (mm)**	HM(-)	7.68 (7.59 to 7.72)	7.62 (7.54 to 7.69)	<0.0001	−0.06 (−0.08 to −0.03)	−0.8 (−1.1 to −0.5)
	HM(+)	7.580 (7.483 to 7.834)	7.575 (7.378 to 7.733)	0.0017	−0.06 (−0.11 to −0.02)	−0.8 (−1.4 to −0.2)
	*P*-value^†^	0.68	0.61		0.77	0.80

IOP, intraocular pressure; AL, axial length; CT, choroidal thickness; CCT, central corneal thickness; K, corneal radius

HM, hypotony maculopathy

* Wilcoxon test for pre-and postoperative data comparisons within groups

^†^Mann-Whitney test for comparisons between groups.

Values represented as median (95% confidence interval for the median).

**Fig 1:**
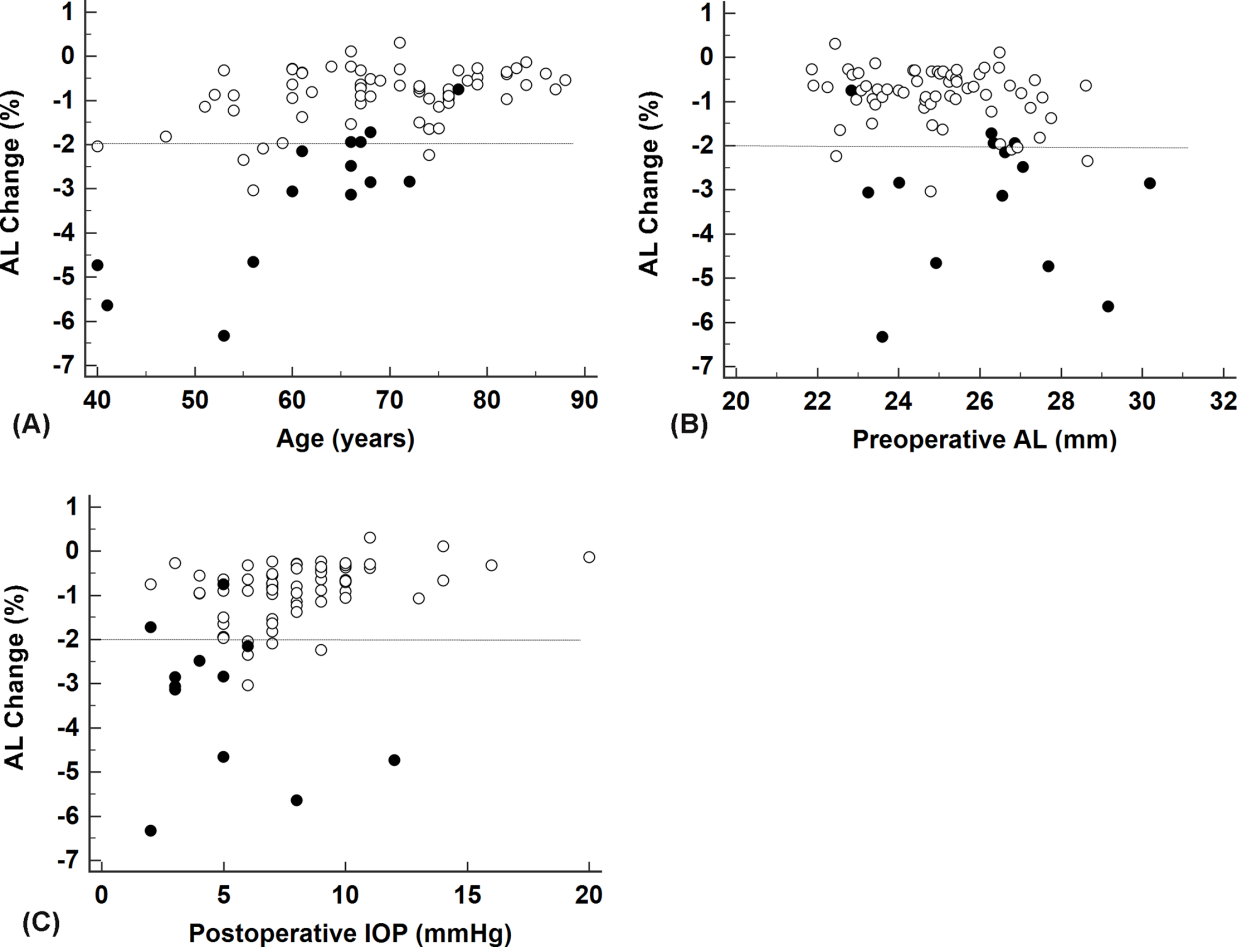
**Scatter plots of percent change of AL with (A) age, (B) preoperative AL, and (C) postoperative intraocular pressure.** AL, axial length; IOP, intraocular pressure. Closed circles indicate eyes with hypotony maculopathy, whereas open circles indicate those without hypotony maculopathy. Dotted lines indicate a cut-off line of 2% reduction rate of AL.

Tables 3 and 4 show Spearman’s correlation coefficients between percent change in AL and the indicated variables, and between percent change of CT and the indicated variables. Fig 1 shows scatter plots of percent AL change against age, preoperative AL, and postoperative IOP. AL reduction rates were positively correlated with age and postoperative IOP, and negatively correlated with preoperative AL. However, Fig 1 also presents how 10 of 14 eyes with HM showed 2% or more AL reduction, whereas five of 63 eyes without HM did so.

**Table 3:** Correlation between the percent change of AL and other parameters.

	Rs	*P*-value
**Age**	0.41 (0.20 to 0.58)	0.0002
**Preoperative AL**	−0.28(−0.48 to −0.06)	0.0132
**Preoperative IOP**	−0.11 (−0.32 to 0.12)	0.36
**Preoperative CCT**	0.06 (−0.17 to 0.28)	0.63
**Postoperative IOP**	0.48 (0.28 to 0.63)	<0.0001
**Percent change of IOP**	0.40 (0.19 to 0.57)	0.0004
**Percent change of CT**	−0.15 (−0.36 to 0.08)	0.20

Rs, Spearman's coefficient of rank correlation; AL, axial length; IOP, intraocular pressure; CCT, central corneal thickness; CT, choroidal thickness.

**Table 4:** Correlation between the percent change of CT and other parameters.

	Rs	*P*-value
**Age**	0.05 (−0.18 to 0.28)	0.65
**Preoperative AL**	0.07 (−0.16 to 0.29)	0.54
**Preoperative IOP**	0.10 (−0.13 to 0.31)	0.41
**Preoperative CCT**	−0.05 (−0.27 to 0.18)	0.68
**Postoperative IOP**	−0.14 (−0.35 to 0.09)	0.25
**Percent change of IOP**	−0.17 (−0.38 to 0.06)	0.15
**Percent change of AL**	−0.15 (−0.36 to 0.08)	0.20

Rs, Spearman's coefficient of rank correlation; CT, choroidal thickness; AL, axial length; IOP, intraocular pressure; CCT; central corneal thickness.

Next, to clarify the influence of AL reduction on development of HM, we set the cut-off value for rate of AL reduction as 2%, and divided subjects into two groups. Table 5 shows between-group data comparisons. Subjects whose AL shortened more than 2% after TLE were significantly younger, and showed longer preoperative AL, than the other group. Preoperatively, there were no differences in CT, central corneal thickness, and corneal radius between the two groups. Postoperative IOP was significantly lower, and IOP rate of change was significantly larger, in eyes where AL reduction rate was ≥2%, than in those where it was <2%. The rate of change of CT, central corneal thickness, and corneal radius did not differ between the groups.

**Table 5:** Comparison of indicated variables of two groups.

AL reduction rate	≥2% (n = 15)	< 2% (n = 62)	*P*-value
Age*, year	57.7±11 (40–74)	70.0±10 (47–88)	<0.0001
**Gender**^**†**^**, Female/Male**	7/8	28/34	1.00
**HM**^**†**^**, (+)/(-)**	10/5	4/58	<0.0001
**Preoperative AL (mm)**^**††**^	26.6 (24.2 to 27.5)	24.9 (24.4 to 25.4)	0.035
**Preoperative CT (μm)**^**††**^	190 (134 to 256)	155 (141 to 184)	0.29
**Preoperative CCT (μm)**^**††**^	515 (467 to 536)	512 (499 to 527)	0.93
**Preoperative K (mm)**^**††**^	7.7 (7.5 to 7.8)	7.7 (7.6 to 7.7)	0.76
**Postoperative IOP (mmHg)**^**††**^	6.0 (3.3 to 6.7)	7.5 (7.0 to 8.2)	0.0062
**Change of IOP (%)**^**††**^	−68.4 (−84.2 to −62.5)	−57.1 (−62.0 to −54.4)	0.0085
**Change of CT (%)**^**††**^	11.7 (3.7 to 30.0)	22.2 (16.7 to 25.7)	0.25
**Change of CCT (%)**^**††**^	2.4 (−3.8 to 3.2)	0.4 (−1.3 to 1.5)	0.82
**Change of K (%)**^**††**^	−1.2 (−1.6 to −0.4)	−0.8 (−1.0 to −0.4)	0.16

AL, axial length; CT, choroidal thickness; CCT, central corneal thickness; K, corneal radius; IOP, intraocular pressure

* Independent sample t-test; data, mean ± standard deviation (range)

^†^ Fisher's exact test

^††^Mann-Whitney test; data, median (95% Confidence interval for the median)

To adjust for age, sex, preoperative AL, postoperative IOP, and change in CT, we calculated propensity scores using a bivariate logistic regression model, by setting the AL reduction rate (>2% or ≤ 2%) as a dependent variable, and sex, age, preoperative AL, postoperative IOP, and change in CT as independent variables. The medians (95% confidence interval) of the propensity scores of eyes with ≥2% AL reduction rate and those with <2% AL reduction rate were 0.81 (0.42–0.91) and 0.008 (0.001–0.019), respectively.

Next, by setting the existence of HM as a dependent variable and the AL reduction rate (≥2% or <2%) as independent variables, bivariate logistic regression with an inverse probability weighting, based on the calculated propensity scores, was conducted using robust variance in the generalized estimating equation model package of SPSS. The odds ratio of eyes with AL reduction rate ≥ 2% for the development of HM was estimated to be 11.67 (95% interval confidence, 1.28–106.6; P-value, 0.03), compared to those with an AL reduction rate <2%. Thus, excessive shortening of AL was significantly associated with HM even after controlling for age, preoperative AL, postoperative IOP, and postoperative CT thickening. This supports the assertion that scleral wall shrinkage contributes to the development of HM after TLE.

## Discussion

The present study demonstrated that IOP reduction, induced by TLE, influenced ocular biometrics. In other words, AL decreased, CT was thickened, and the radius of corneal curvature decreased after TLE, whereas no changes occurred in CCT, irrespective of the presence of HM. These findings agree with previous reports [5–10, 13]. AL reduction after TLE, measured by IOLMaster, was 0.1–0.32 mm [5–7, 13]. In the previous study, we reported AL reduction (reduction rate) as 1.59 mm (5.9%) and 0.38 mm (1.5%) for subjects with and without HM, respectively. Here all subjects had IOP ≤6 mmHg at 4 weeks post TLE [11]. In this study, the magnitude and rate of AL reduction, at the time of trough IOP, were 0.7 mm (2.8%) and 0.18 mm (0.7%) for subjects with and without HM, respectively. Both of these variables were significantly larger in eyes with HM, compared to eyes without HM, as previously reported.

Bivariate logistic regression analysis with inverse probability weighting, revealed that (even after controlling for known risk factors for the development of HM such as sex, age, preoperative AL, postoperative IOP, and the change of CT), AL reduction rates ≥ 2% had 11.7 times more risk of developing HM.

The choroid is thickened and IOP is lowered after TLE. This likely occurs as a result of increases in intravascular volume [5, 6, 10]. Choroidal thickening may influence AL measurement by IOLMaster, because it defines AL as the distance from the surface of the cornea to the pigment epithelium, just above the choroid. In this study, increases in CT were not different between subjects with and without HM, or between eyes of where the AL reduction rate was 2%> or 2%≤. Change in CT was around 30μm (0.03 mm), accounting for only approximately 17% of AL change. Therefore, there was limited influence of choroidal thickening on AL reduction after TLE. Considering the high association between AL reduction and HM, it is reasonable to state that scleral wall shrinkage, rather than choroidal thickening, contributes to development of HM.

In the current study, average corneal curvature radius decreased after TLE, irrespective of the presence or absence of HM. Kook et al. reported steepening of overall vertical corneal curvature after TLE, concluding it could be due to tight scleral flap sutures and a “posteriorly placed wound gape” from internal sclerotomy [14]. Although we did not analyze separate changes in corneal curvature, along the horizontal and vertical axis, or its relationship with scleral flap sutures, these findings indicate that the cornea was not flattened, even when IOP decreased after TLE. This lends further support to the idea that structural changes in the posterior eye were responsible for AL shortening.

Previous studies found that central corneal thickness did not change after TLE [13, 15], just as in this study. As for the corneal thickness, Nicolela et al. studied 13 patients with HM and 25 controls with hypotony. They found that eyes with HM had significantly thicker corneas than controls, and concluded that patients with hypotony and thinner central corneas were at lower risk for HM [16]. Because IOP measurements in eyes with thicker corneas may be overestimated, true postoperative IOP in eyes with thicker corneas may be lower than measured. Nicolela et al. speculated that such a measurement artifact could account for seemingly higher risk for HM in the eyes with thicker corneas. In the current study, there were no differences, in either corneal curvature or central corneal thickness, pre- and postoperatively, between the eyes with and without HM.

Contrary to the previous reports, the groups did not differ with regard to gender in this or our previous study. The eyes of male subjects are bigger than females; i.e., males have longer AL compared to females. This may account for previous findings that male gender was a risk factor for developing HM. In the current study, preoperative AL in males and females were 25.5 ± 1.7 mm and 24.7 ± 1.9 mm, respectively. Although males had slightly longer ALs, the difference did not rise to the level of statistical significance (p = 0.059, independent samples t-test).

Suner et al. found that HM occurred more frequently after laser suture lysis, bleb leaks, and needling procedures[17]. In this study, however, the incidence of these events did not differ between the groups, indicating these events alone did not increase the risk for HM.

Gass noted that choroidal detachment seldom occurs in eyes with HM, and may even be a protective mechanism against HM [3]. However, the incidence of choroidal effusion did not differ between subjects with and without HM, in this study. Some patients exhibited both HM and choroidal effusion, either simultaneously or at different time points. The difference in detection rate of HM between Gass and this study may be due to differences in methodology. Gass defined HM solely by fundscopy, whereas we used OCT, probably increasing the likelihood that HM was detected. Further studies are needed to explore the relationship between choroidal detachment and HM.

There are several limitations in this study. The sample size, particularly of the eyes with HM, was insufficient for multivariate logistic regression analysis using raw data sets. However, bivariate logistic regression analysis, using an inverse probability weighting based on the propensity scores to adjust for sex, age, preoperative AL, postoperative IOP, and change of CT, revealed that the AL reduction rates ≥ 2% correlated with development of HM after TLE. AL measurements and OCT imaging were difficult to perform in the early days after TLE. This may have produced an inclusion bias. In addition, we did not consider diurnal fluctuation in CT during this study. However, the magnitude of such a fluctuation is reported to be at most 33 μm [7]. In comparison, the magnitude of AL change was 0.18 mm (180 μm) in the eyes without HM and 0.7mm (700 μm) in the eyes with HM. Therefore, we believe that the effect of diurnal fluctuation of the choroidal thickness is minimal, if any.

In conclusion, we confirmed that the amount and rate of AL shortening, induced by IOP reduction after TLE, was greater in eyes with HM than those without HM. Given that the CT did not differ pre- to postoperatively in eyes with and without HM, and that eyes with the AL reduction rates >2% had significantly higher risk of developing HM, even after controlling for other parameters, this study supports Gass’ theory that scleral biomechanical properties and scleral shrinkage primarily contribute to the development of hypotony maculopathy after TLE.

## Supporting information

S1 Dataset The raw data of all subjects. (XLSX)

S1 File Certificate of editing. (PDF)
