# Supplementary material for: Excessive scleral shrinkage, rather than choroidal thickening, is a major contributor to the development of hypotony maculopathy after trabeculectomy
Source: PLoS One. 2018 Jan 26;13(1):e0191862. doi: 10.1371/journal.pone.0191862 (PMC5786308; doi:10.1371/journal.pone.0191862)
Supplement: S1 File — (PDF) [file pone.0191862.s002.pdf]

enago www.enago.jp Author First, Quality First 英文校正・校閲-エナゴ | ulatus www.ulatus.jp 論文翻訳・学術翻訳-ユレイタス | voxtab www.voxtab.jp 英語テープ起こし-ボックスタブ

CERTIFICATE OF EDITING

This is to certify that the paper titled Excessive scleral shrinkage, rather than choroidal thickening, is a major contributor to the development of hypotony maculopathy after trabeculectomy commissioned to us by Makoto Nakamura (神戸大学) has been edited for English language, grammar, punctuation, and spelling by Enago, the editing brand of Crimson Interactive Pvt. Ltd under Advance Editing.

Issued by:
Enago, Crimson Interactive Pvt. Ltd.
1001, Techniplex - II, S. V. Road,
Goregaon (W), Mumbai 400062, India.
Phone: 03-5050-5374
Fax: 03-4496-4934

Disclaimer : The author is free to accept or reject our changes in the document after our editing. However, we do not bear responsibility for revisions made to the document after our edit on 16 Jun 2017.

**Global**	www.enago.com, www.ulatus.com, www.voxtab.com	**Germany**	www.enago.de
**Japan**	www.enago.jp, www.ulatus.jp, www.voxtab.jp	**Russia**	www.enago.ru
**Taiwan**	www.enago.tw	**Arabic**	www.enago.ae
**China**	www.enago.cn	**Turkey**	www.enago.com.tr
**Brazil**	www.enago.com.br	**S. Korea**	www.enago.co.kr

About Crimson:
Crimson Interactive Inc. provides English language editing, transcription, and translation services to individuals and corporate customers worldwide.
